# Long-term outcome of percutaneous transhepatic venous embolization for ectopic peristomal variceal bleeding: A case report

**DOI:** 10.1097/MD.0000000000050706

**Published:** 2026-09-11

**Authors:** Jie Yuan, Tian Zhao, Huanling Zeng

**Affiliations:** a Department of Radiology, Sir Run Run Shaw Hospital, Zhejiang University School of Medicine, Hangzhou, Zhejiang, China; b Xiaolan Clinical Institute of Shantou University Medical College, Zhongshan, Guangdong, China; c Department of Oncology, Xiaolan People’s Hospital of ZhongShan (The Fifth People’s Hospital of ZhongShan), Zhongshan, Guangdong, China.

**Keywords:** case report, ectopic varices, percutaneous transhepatic obliteration, peristomal varices, portal hypertension

## Abstract

**Rationale:**

Bleeding from ectopic varices secondary to portal hypertension represents a significant therapeutic challenge due to the absence of standardized management protocols.

**Patient concerns:**

This case report details the long-term management and follow-up of a rare and challenging case of recurrent hematuria caused by bleeding from ectopic peristomal varices secondary to portal hypertension in a patient with an ileal neobladder.

**Diagnoses:**

Hematuria secondary to ectopic peristomal varices of an ileal neobladder associated with portal hypertension.

**Interventions:**

The patient underwent 2 sessions of percutaneous transhepatic obliteration (PTO) (in April 2018 and February 2022) for active variceal bleeding.

**Outcomes:**

After the initial PTO, hematuria resolved immediately, and a follow-up computed tomography at 3 months confirmed complete occlusion of the variceal complex. Recurrent hematuria occurred at 16 and 20 months post-procedure; imaging demonstrated recanalization attributable to persistent portal hypertension rather than technical failure of the initial embolization. A second PTO in February 2022 again achieved complete control of bleeding. No further hematuria occurred after the second PTO, for a recurrence-free interval of approximately 3 years and 10 months (February 2022 to December 2025), approximately 7 years and 8 months after the initial procedure. The most recent computed tomography revealed only minimal, nonprogressive residual peristomal varices without active bleeding.

**Lessons:**

PTO may provide immediate hemostasis and durable symptom control in selected patients with ectopic peristomal variceal bleeding; however, because embolization does not treat the underlying portal hypertension, recurrence remains possible, necessitating structured long-term surveillance and readiness for repeat intervention or portal pressure–reducing therapy. This case, with 7-year follow-up, illustrates the feasibility and long-term outcomes of repeated PTO for ectopic variceal bleeding at a complex anatomical site.

## 1. Introduction

Ectopic varices are portosystemic collaterals that develop outside the gastroesophageal region, most commonly as a consequence of portal hypertension, portal vein thrombosis, or prior abdominal surgery.^[1,2]^ They account for 1 to 5% of all variceal bleeding episodes and are associated with considerable mortality.^[2]^ Owing to their rarity and heterogeneous anatomy, no standardized management protocol for bleeding ectopic varices has been established; current evidence derives predominantly from isolated case reports, small case series, and expert consensus.^[2–4]^

Current management strategies encompass endoscopic therapy, surgical ligation, and endovascular intervention. Endovascular techniques are increasingly favored for extragastrointestinal sites that are inaccessible to endoscopy, and principally include percutaneous transhepatic obliteration (PTO)/percutaneous antegrade transhepatic venous obliteration (PATVO), balloon-occluded retrograde transvenous obliteration (BRTO) and its modifications (plug-assisted and coil-assisted retrograde transvenous obliteration), and transjugular intrahepatic portosystemic shunt (TIPS).^[3–7]^ International guidelines and consensus documents, including the Baveno VII consensus and the American Association for the Study of Liver Diseases practice guidance, emphasize that any local hemostatic therapy should be integrated into the comprehensive management of the underlying portal hypertension.^[8,9]^

Several case reports and small series have described PTO for ectopic varices; however, long-term outcome data are essentially absent, and the durability of embolization under persistent portal hypertension remains poorly documented. We therefore report a case of ectopic peristomal variceal bleeding presenting as recurrent hematuria after ileal neobladder reconstruction, managed with 2 sessions of PTO, with emphasis on the 7-year follow-up, the delayed recurrence and repeat embolization, and the durable long-term clinical control achieved under structured surveillance.

## 2. Case presentation

A 66-year-old man was admitted to our hospital on April 4, 2018, with recurrent gross hematuria for more than 4 months, 10 years after radical cystectomy. In 2008, he had undergone radical cystectomy with ileal neobladder reconstruction for an incidentally discovered bladder mass; postoperative pathology confirmed noninvasive papillary urothelial carcinoma, and no recurrence was observed on regular surveillance thereafter. In December 2017, he developed painless light-red bleeding from the urinary stoma. At a local hospital, cystoscopy showed ileal neobladder changes without an identifiable bleeding site, and urine cytology was negative; he was discharged after conservative hemostatic treatment. On March 27, 2018, bright-red gross hematuria recurred without clots.

His past medical history was notable for hepatitis B–related liver cirrhosis of more than 10 years’ duration. He denied long-term alcohol consumption or prior blood transfusion. Physical examination on admission showed a patent urinary stoma in the right lower abdomen draining light-red urine, without abdominal tenderness or rebound tenderness. He had been receiving long-term oral nonselective beta-blocker therapy for portal hypertension.

Urinalysis was positive for occult blood (3+) and protein (3+), with weakly positive leukocytes. Laboratory tests showed hemoglobin 69 g/L, white blood cell count 4.6 × 10^9^/L, platelet count 102 × 10^9^/L, international normalized ratio 1.05, serum albumin 31.8 g/L, total bilirubin 0.6 mg/dL, and C-reactive protein 1.8 mg/L. Liver function was Child–Pugh class A (score 6), with a model for end-stage liver disease score of 8. Serology was positive for hepatitis B core antibody (immunoglobulin G) and negative for hepatitis C antibody. Contrast-enhanced abdominal computed tomography (CT) demonstrated postsurgical changes of the bladder, a right lower abdominal stoma, left-sided hydronephrosis, and prominent tortuous peristomal veins. The liver contour was irregular with lobar redistribution consistent with cirrhosis, accompanied by splenomegaly; no ascites or portal vein thrombosis was present. Taken together, the lesion was defined as an ectopic peristomal varix of the ileal neobladder: a portosystemic collateral complex located in the abdominal wall surrounding the urinary stoma, fed by the ileal branch of the superior mesenteric vein (SMV) and draining through the superficial epigastric vein into the femoral vein.

### 2.1. Preoperative evaluation and treatment planning

Recurrent urothelial carcinoma was excluded by negative urine cytology, surveillance cystoscopy, and imaging. Urinary tract infection was excluded by a normal white blood cell count and C-reactive protein. Nephrolithiasis was excluded on CT, and coagulopathy was excluded by an essentially normal international normalized ratio. Renal parenchymal bleeding was considered unlikely given the absence of renal lesions on contrast-enhanced CT and the characteristic peristomal collateral anatomy on CT angiography. Based on the established history of cirrhosis and the imaging findings, the hematuria was attributed to rupture of the ectopic peristomal varices secondary to portal hypertension. CT angiography confirmed the portosystemic collateral anatomy described above, establishing the indication for intervention.

PTO was selected as the primary strategy after multidisciplinary consultation for the following reasons. It provides direct, antegrade, superselective access to both the feeding vessel and the varix, permitting definitive obliteration at the bleeding site. Unlike BRTO, it does not require a balloon-occludable gastrorenal or splenorenal outflow, which the anatomy in this patient (drainage via abdominal wall collaterals into the femoral vein) did not provide. Unlike TIPS, it preserves portal venous perfusion and carries no additional risk of hepatic encephalopathy, an important consideration given the focal, anatomically accessible nature of the bleeding. TIPS was reserved as a contingency option in case of suboptimal response.

### 2.2. Initial interventional procedure (April 16, 2018)

**Figure 1.**
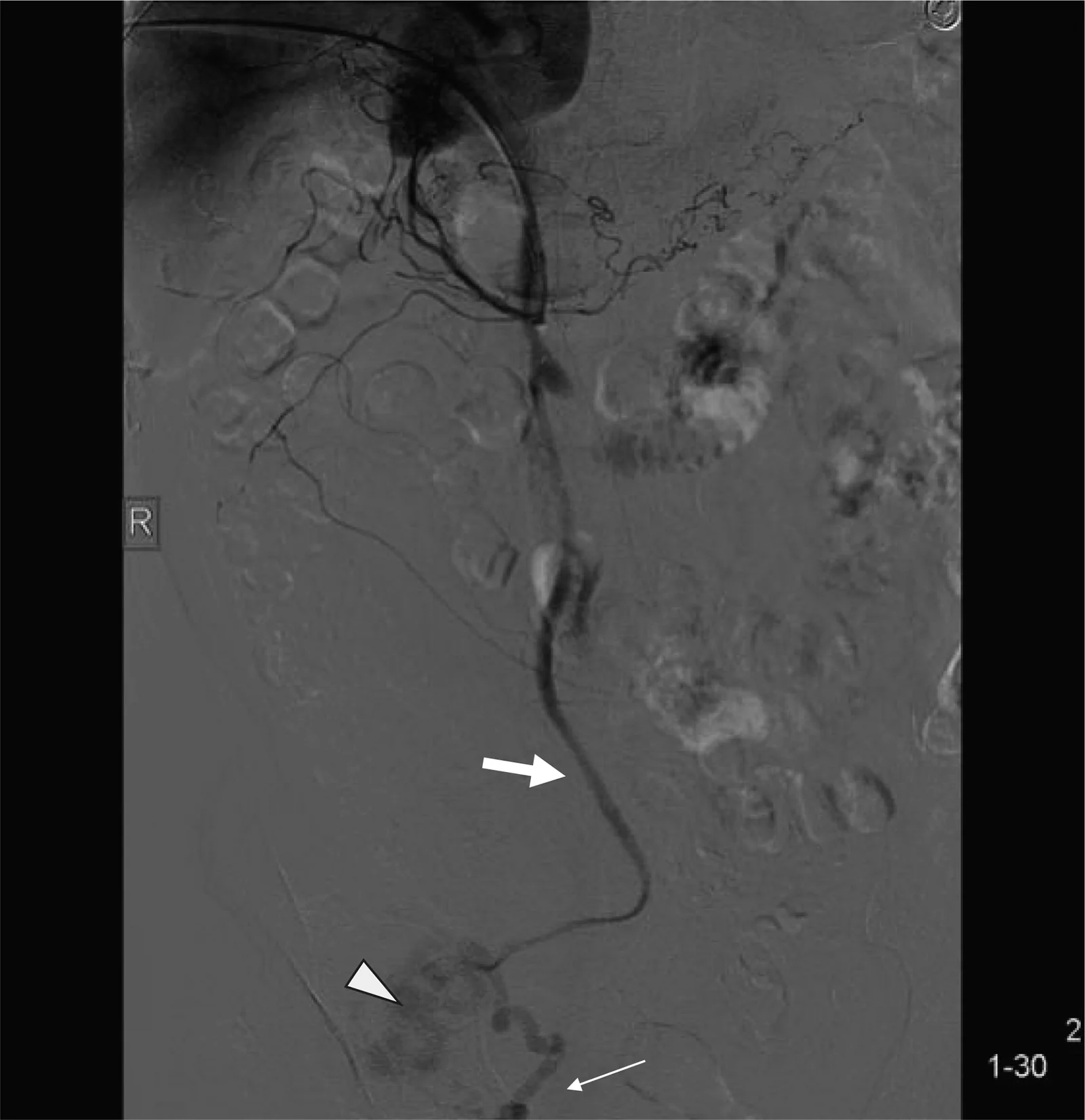
First PTO (April 2018): transhepatic portography showing a markedly tortuous peristomal variceal plexus (thick arrow) supplied by the ileal branch of the superior mesenteric vein (triangle), with a dominant efferent abdominal wall vein draining into the right femoral vein (thin arrow). PTO = percutaneous transhepatic obliteration.

**Figure 2.**
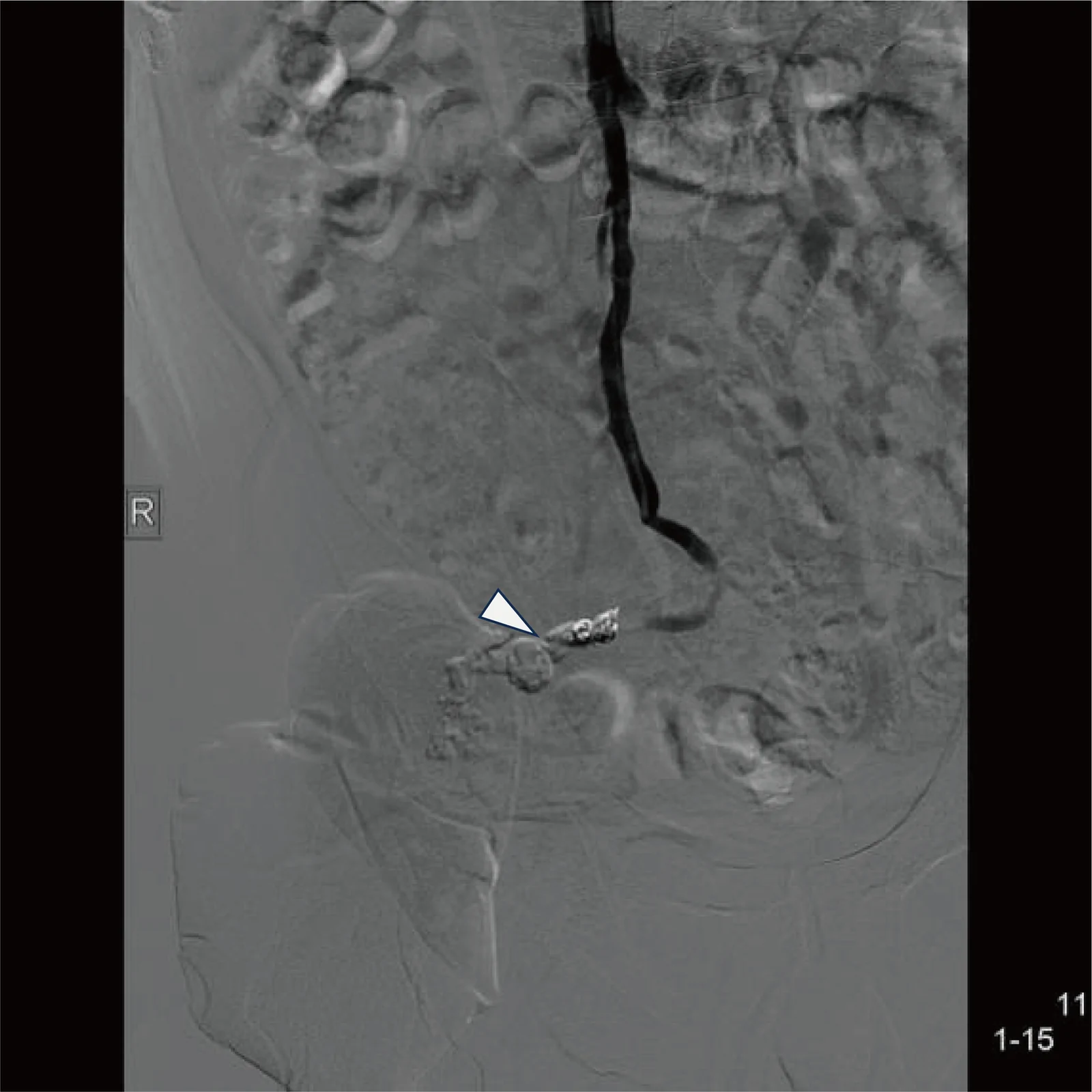
First PTO (April 2018): post-embolization portography confirming complete occlusion of the variceal complex. The NBCA–Lipiodol cast and 2 reinforcing coils (triangle) are shown; the efferent vein is no longer opacified. NBCA = N-butyl-2-cyanoacrylate, PTO = percutaneous transhepatic obliteration.

Under local anesthesia, a peripheral portal venous branch was punctured under ultrasound guidance via a transhepatic approach. Portography with a 5-Fr Tempo angiographic catheter (Cordis) confirmed the portosystemic shunt from the ileal branch of the SMV to the right femoral vein via the peristomal variceal plexus (Fig. 1). The pressure in the SMV was 26 cmH_2_O before embolization. A 2.7-Fr Progreat microcatheter (Terumo) was advanced coaxially over a microwire and positioned superselectively at the orifice of the variceal plexus. Three milliliters of a 40% N-butyl-2-cyanoacrylate–ethiodized oil (Lipiodol) emulsion was injected for embolization, followed by reinforcement with 2 8 mm × 14 cm coils (Cook Medical). Post-embolization angiography confirmed complete occlusion of the variceal complex (Fig. 2), and the SMV pressure rose to 37 cmH_2_O. Hematuria resolved on postoperative day 1. The patient experienced mild pain in the stoma region without other complications and was discharged in improved condition.

### 2.3. Follow-up

The surveillance protocol consisted of outpatient clinical assessment with urinalysis and complete blood count every 3 months during the first year after each procedure and every 6 to 12 months thereafter, and contrast-enhanced abdominal CT at 3 months after each procedure and annually thereafter, or promptly upon any recurrence of hematuria; the patient also continued hepatology follow-up for hepatitis B–related cirrhosis. Follow-up CT at 3 months after the initial procedure showed complete resolution of the variceal complex without evidence of active bleeding. However, recurrent hematuria occurred at 16 and 20 months post-intervention. CT angiography confirmed recanalization of the varices through the previously documented portosystemic shunt pathway. Given the persistent portal hypertension, secondary interventions including BRTO or TIPS were recommended. The patient, whose hemoglobin was stable at 131 g/L, declined further invasive treatment; he received hemostatic pharmacotherapy and was discharged with symptomatic improvement.

### 2.4. Re-intervention

**Figure 3.**
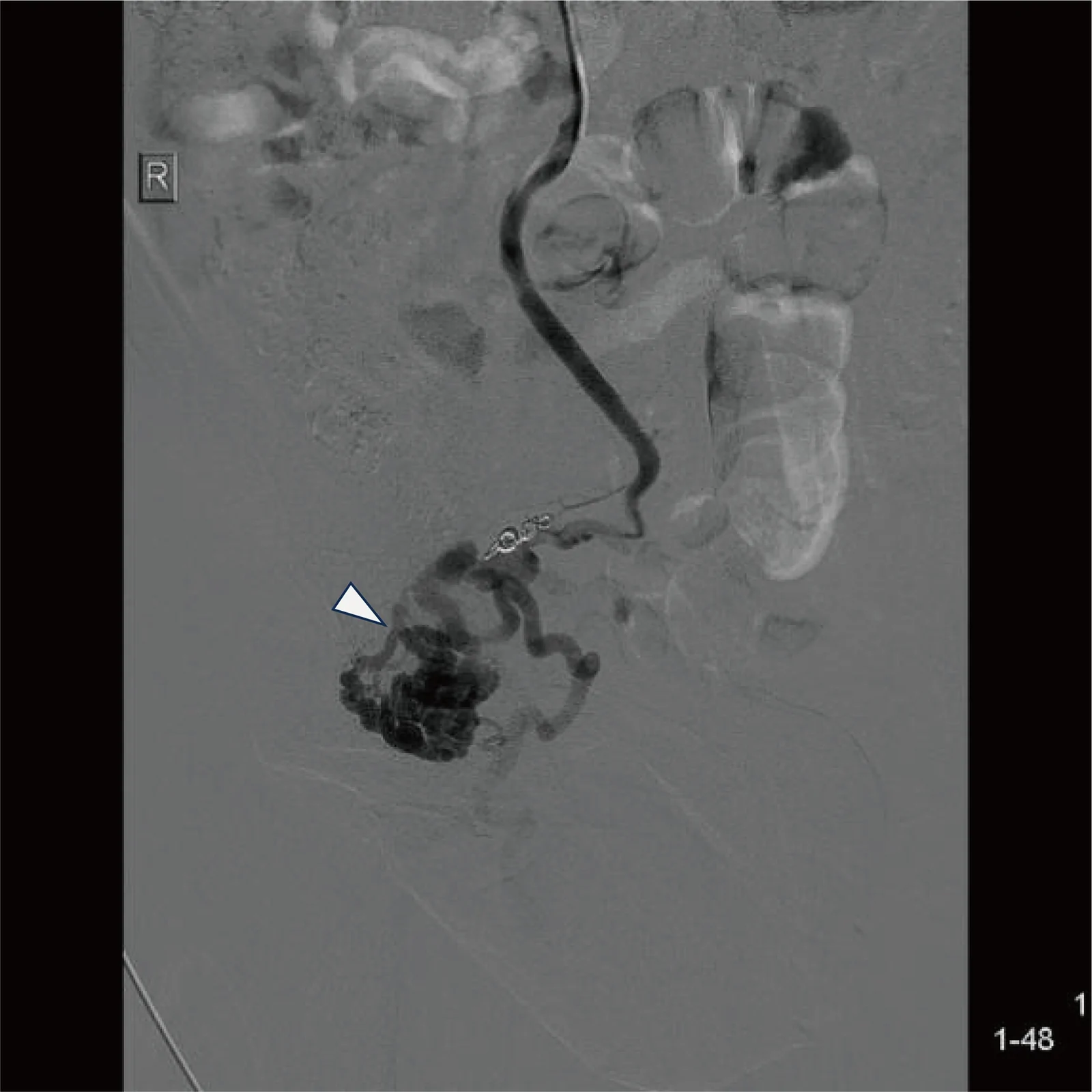
Second PTO (February 2022): portography before repeat embolization showing recanalized, enlarged, tortuous peristomal varices (triangle) around the urinary stoma, opacified via the same portosystemic shunt pathway. PTO = percutaneous transhepatic obliteration.

**Figure 4.**
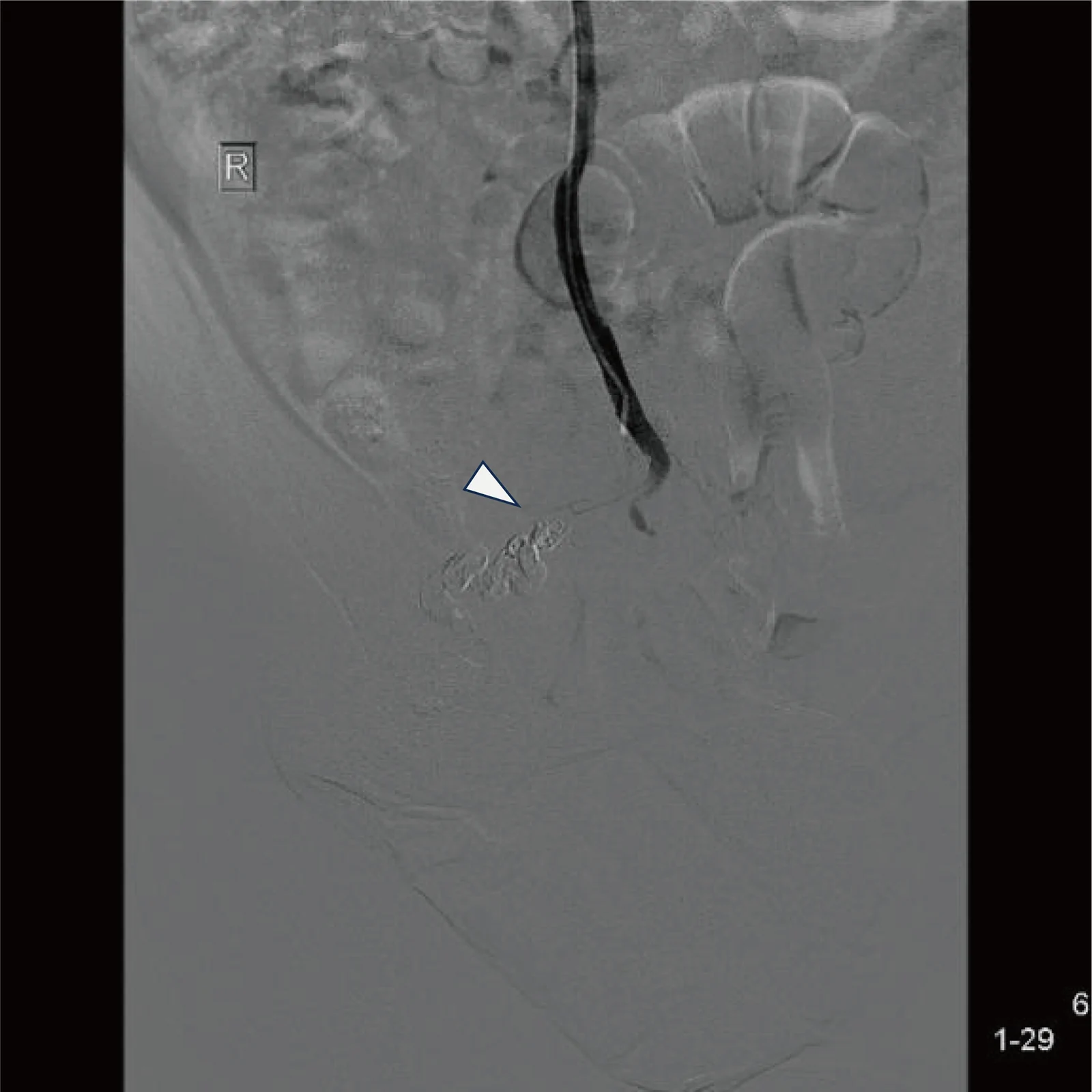
Second PTO (February 2022): post-embolization angiography showing no residual opacification of the variceal plexus (triangle indicates the embolization site with coil cast). PTO = percutaneous transhepatic obliteration.

In February 2022, the patient was readmitted with 1 day of gross hematuria. Physical examination revealed a patent urinary stoma in the right lower abdomen with a small amount of bloody urine. Contrast-enhanced abdominal CT demonstrated enlarged, tortuous peristomal veins. Hemoglobin was 126 g/L on admission, decreasing to 96 g/L 5 hours later. A second PTO was performed using the same technique to embolize the recanalized variceal complex (Figs. 3 and 4). Hematuria resolved completely after the intervention; the patient reported only mild stoma-site pain without other complications.

### 2.5. Long-term follow-up

**Figure 5.**
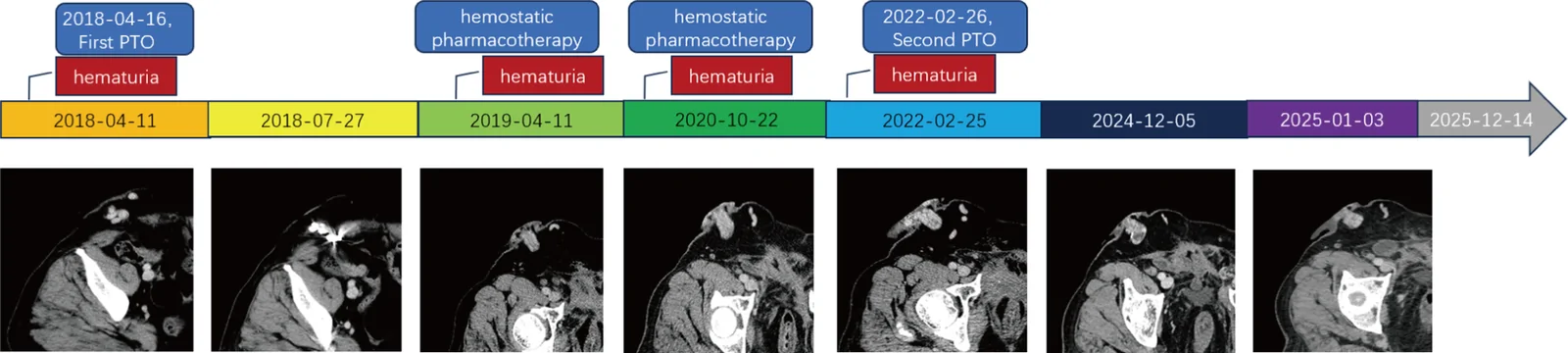
Long-term follow-up: contrast-enhanced CT obtained at the 7-year follow-up showing minimal, nonprogressive residual peristomal varices without active bleeding. The timeline above the images indicates the key clinical events: first PTO (April 2018), recurrences (16 and 20 months), second PTO (February 2022), and the 7-year follow-up CT (December 2025). CT = computed tomography, PTO = percutaneous transhepatic obliteration.

The patient was monitored through scheduled clinical and imaging evaluations until December 14, 2025, a follow-up duration of 7 years and 8 months after the initial PTO. No further hematuria occurred after the second PTO, for a recurrence-free interval of approximately 3 years and 10 months (February 2022 to December 2025). Follow-up contrast-enhanced CT performed 2 years after the second intervention showed minimal, nonprogressive residual peristomal varices without radiologic evidence of active bleeding (Fig. 5). The clinical course is summarized in Table 1.

**Table 1:** Clinical course of the patient.

Date	Clinical event	Hemoglobin (g/L)	Key imaging findings	Intervention/outcome
Dec 2017	First episode of painless stoma bleeding	102	Cystoscopy: ileal neobladder changes, no bleeding site; urine cytology negative	Conservative hemostatic treatment
Apr 2018	Active variceal bleeding	69	Portography: SMV ileal branch → peristomal variceal plexus → right femoral vein; SMV pressure 26 cm H_2_O	First PTO (NBCA–Lipiodol + 2 coils); hematuria resolved on day 1
Jul 2018 (3 mo post-op)	Asymptomatic	129	CT: complete occlusion of variceal complex	Routine surveillance
Aug–Dec 2019 (16–20 mo)	Recurrent hematuria × 2 episodes	131 (stable)	CTA: recanalization via the same shunt pathway	PTO/TIPS recommended, declined; hemostatic pharmacotherapy
Feb 2022	Readmission for gross hematuria (1 day)	126 → 96 (5 h)	CT: enlarged tortuous peristomal veins	Second PTO; hematuria resolved
Dec 2025 (7 yr)	No recurrence of hematuria	135	CT (2 yr after 2nd PTO): minimal, nonprogressive residual peristomal varices, no active bleeding	Continued surveillance

CT = computed tomography, CTA = computed tomography angiography, NBCA = N-butyl-2-cyanoacrylate, PTO = percutaneous transhepatic obliteration, SMV = superior mesenteric vein, TIPS = transjugular intrahepatic portosystemic shunt.

## 3. Discussion

This case illustrates the long-term course of recurrent hematuria caused by ruptured ectopic peristomal varices secondary to portal hypertension in a patient with an ileal neobladder, managed with 2 PTO procedures and followed for 7 years. Rather than the embolization technique itself, the principal contribution of this report is the exceptionally long follow-up, which captured 3 clinically instructive findings. First, although PTO achieved immediate hemostasis on both occasions, imaging surveillance documented delayed recanalization with recurrent bleeding at 16 to 20 months after the first procedure, underscoring that embolization alone does not address the underlying portal hypertension, and that only extended follow-up can reveal such delayed recurrence. Second, even after the second intervention, small residual varices were present on imaging 2 years later yet remained asymptomatic, reinforcing that anatomical closure does not equate to hemodynamic cure and that surveillance should continue despite clinical quiescence. Third, the absence of rebleeding over the nearly 4 years since the second PTO suggests that, in this patient, timely repeat embolization combined with structured monitoring achieved durable clinical control despite residual or recanalized vasculature.

The unusual presentation of hematuria (rather than the gastrointestinal bleeding typical of ectopic varices) can be explained by the surgical anatomy. Radical cystectomy with ileal neobladder reconstruction creates postoperative adhesions that bring the mesenteric venous territory (the ileal branch of the SMV) into apposition with the rich systemic venous network of the anterior abdominal wall. Under sustained portal hypertension, portosystemic collaterals develop across these adhesions; the resulting variceal plexus lies within the stomal and peristomal tissues, immediately adjacent to the urothelium-lined neobladder and its cutaneous stoma, so that rupture produces bleeding through the urinary stoma (hematuria) rather than gastrointestinal hemorrhage. This mechanism is consistent with published reports of peristomal variceal hemorrhage after ileal conduit or neobladder urinary diversion.^[4–7,10,11]^

Ectopic variceal bleeding is a rare yet potentially life-threatening entity. A synthesis of prior case reports delineates 3 characteristic features: underlying portal hypertension, a history of abdominal surgery, and presentation with bleeding at atypical sites.^[3–7]^ Postoperative adhesions may facilitate aberrant portosystemic collaterals between the mesenteric venous system and the systemic veins of the abdominal wall, as in the present case.

Current management strategies for ectopic varices encompass endoscopic therapy, surgical ligation, and endovascular intervention.^[3]^ Given the anatomical constraints of endoscopic access to extragastrointestinal sites, endovascular techniques have emerged as a principal option. Transcatheter embolization can be performed via several routes, including PTO/PATVO,^[12]^ BRTO,^[13]^ and TIPS.^[14]^ Alternative approaches described in case reports include embolization through a recanalized paraumbilical vein using N-butyl-2-cyanoacrylate^[15]^ and direct percutaneous embolization with gelatin sponge particles and ethanolamine oleate iopamidol.^[16]^ Each method, alone or in combination, presents distinct limitations, risks, and benefits.

A representative series of 12 patients undergoing 17 sessions of PATVO for active parastomal or small-bowel variceal bleeding reported a technical success of 100% (17/17) and an early clinical success of 82.3% (14/17).^[12]^ The principal advantage of the antegrade transhepatic approach is a direct, precise, and minimally invasive route, particularly valuable for focal varices with an identifiable feeding vessel, as in our patient. BRTO has likewise proven effective for ectopic varices, including those of mesenteric origin: Koo et al,^[17]^ Ikeda et al,^[18]^ Ono et al,^[19]^ and Sato et al^[20]^ each reported successful retrograde obliteration via abdominal wall collateral pathways. Modified BRTO techniques (plug-assisted and coil-assisted retrograde transvenous obliteration) have shown favorable outcomes for ectopic variceal hemorrhage.^[11,21,22]^ In a series of 15 duodenal varices cases, BRTO achieved curative obliteration in 8 of 9 treated cases, while PTO served as primary therapy in 3 cases in which anatomical constraints precluded BRTO, with eradication in all 3; highlighting that the choice between PTO and BRTO should be individualized to the vascular anatomy.^[13]^

TIPS is a well-established intervention for variceal hemorrhage and generally offers lower long-term rebleeding rates, particularly in diffuse portal hypertension; however, it carries notable risks, principally stent dysfunction and hepatic encephalopathy. A systematic review of TIPS for ectopic variceal bleeding reported complications in 42% of 15 patients, including rebleeding and hepatic encephalopathy.^[14,23]^ Compared with TIPS, PTO is less technically demanding and less invasive, and preserves portal perfusion.^[24,25]^

The choice of repeat PTO rather than TIPS after recurrence warrants explanation. The recurrence remained focal and was again accessible via the same antegrade route, making repeat embolization technically straightforward; the patient had preserved liver function (Child–Pugh A), and the bleeding was focal rather than diffuse, so the encephalopathy risk and the commitment to lifelong shunt surveillance associated with TIPS were not considered justified; and the patient had previously declined more invasive options. TIPS nevertheless remains the definitive portal pressure–reducing strategy and is our stated escalation option should bleeding recur and can be combined with PTO when necessary.

Management of the underlying portal hypertension is integral to the care of such patients. Our patient has remained under hepatology follow-up for hepatitis B–related cirrhosis. Consistent with the Baveno VII consensus and American Association for the Study of Liver Diseases guidance, nonselective beta-blockade and definitive portal pressure–reducing strategies (e.g., TIPS) should be considered as part of comprehensive management and as escalation options in the event of further recurrence.^[8,9]^ Embolization obliterates the local varix but does not reduce portal pressure; persistent portal hypertension promotes both recanalization of the embolized channel and recruitment of new collaterals across postoperative adhesions; accounting for the recanalization demonstrated at 16 to 20 months and the small residual varices seen at late follow-up in this case.

This report has limitations. It describes a single patient, and the durability of PTO observed here may not be generalizable to patients with more advanced liver disease, higher portal pressure gradients, or different collateral anatomy. Portal pressure was not remeasured during follow-up, and surveillance intervals were pragmatic rather than protocolized. Finally, portal pressure–reducing therapy was not instituted, so the comparative effectiveness of PTO versus TIPS cannot be inferred from this case.

In conclusion, in selected patients with focal, anatomically accessible ectopic peristomal variceal bleeding, PTO may provide immediate hemostasis and durable symptom control. Because embolization does not treat the underlying portal hypertension, structured long-term clinical and imaging surveillance (and readiness to repeat embolization or escalate to portal pressure–reducing therapy) are essential.

## Author contributions

**Conceptualization:** Jie Yuan, Tian Zhao.

**Data curation:** Jie Yuan, Tian Zhao, Huanling Zeng.

**Formal analysis:** Jie Yuan, Huanling Zeng.

**Investigation:** Jie Yuan, Tian Zhao, Huanling Zeng.

**Methodology:** Jie Yuan, Tian Zhao, Huanling Zeng.

**Writing – original draft:** Jie Yuan, Tian Zhao, Huanling Zeng.

**Writing – review & editing:** Jie Yuan, Tian Zhao, Huanling Zeng.
